# Case Report: Urgent ultrasound-guided dual-lumen PICC placement after platelet transfusion in severe aplastic anemia with extreme thrombocytopenia

**DOI:** 10.3389/fmed.2026.1915465

**Published:** 2026-08-28

**Authors:** Yeyan He, Shasha Li, Yimei Feng, Xiaoli Chen, Xi Zhang

**Affiliations:** Medical Center of Hematology, The Second Affiliated Hospital of Army Medical University, Chongqing, China

**Keywords:** case report, hematopoietic cell transplantation, peripherally inserted central catheter, severe aplastic anemia, thrombocytopenia

## Abstract

**Background:**

Patients with severe aplastic anemia (SAA) proceeding to allogeneic hematopoietic cell transplantation (HCT) require reliable central venous access, yet the procedural evidence base was generated at platelet counts of roughly 10–50 × 10^9^/L and does not extend to the extreme thrombocytopenia of marrow failure.

**Case description:**

A 56-year-old woman with SAA, neutropenia, fungal maxillary sinusitis, scattered ecchymoses, and no active spontaneous bleeding required urgent multi-lumen access before conditioning. Her admission platelet count was 1 × 10^9^/L, and after one 200-mL apheresis platelet transfusion the count measured approximately 14 h later was 2 × 10^9^/L, a poor sustained increment. Because that sample fell outside any standardized post-transfusion interval, a corrected count increment could not be calculated and refractoriness could not be established; HLA and HPA antibody testing was not available within the procedural window.

**Intervention and outcomes:**

After multidisciplinary review, a second 200-mL apheresis platelet transfusion was given and a 5-Fr dual-lumen power-injectable PICC was inserted immediately afterward through the right basilic vein under ultrasound guidance. Insertion followed the transfusion without an interval, so the last recorded count of 2 × 10^9^/L precedes the second unit and the value at venipuncture was not remeasured. The catheter-to-vein ratio, measured from the stored procedural images, was approximately 32%. Access was achieved on the first puncture and the tip confirmed in the lower third of the superior vena cava. World Health Organization grade 1 puncture-site oozing stopped after 10 min of compression. No bleeding of grade 2 or higher, transfusion reaction, occlusion, migration, catheter-related bloodstream infection, or clinically evident catheter-related thrombosis was documented over 63 catheter-days or at 3-month follow-up. Routine thrombosis-screening ultrasound was not performed.

**Conclusion:**

This report documents how a coordinated risk-control pathway combining necessity assessment, platelet support, a compressible access site, first-pass ultrasound-guided insertion, and interval-specific surveillance was applied when transplantation could not be deferred, and it specifies the measurements that future prospective series should capture. A single case cannot establish the safety of PICC placement at extremely low platelet counts or define a platelet threshold, particularly because the count at the time of insertion was not remeasured.

## Introduction

Severe aplastic anemia (SAA) is a life-threatening marrow-failure syndrome characterized by hypocellular marrow, pancytopenia, infection susceptibility, and spontaneous or procedure-associated bleeding. Timely allogeneic hematopoietic cell transplantation (HCT) may be definitive for eligible patients, but the supportive procedures required to begin it can themselves become high-risk decisions (1–4).

Central venous access is indispensable for conditioning chemotherapy, blood products, anti-infective therapy, and parenteral support. In profound thrombocytopenia the question is not whether a catheter can be inserted but whether the consequences of delay and of repeated peripheral venipuncture outweigh the risks of bleeding, hematoma, infection, thrombosis, and device failure, risks modified by site compressibility, operator experience, ultrasound guidance, and catheter characteristics.

Current platelet-transfusion guidance supports context-sensitive decision-making for catheter placement, but the randomized evidence was generated at counts of roughly 10 to 50 × 10^9^/L (5, 6), and PICC series in hematology and oncology, although reporting low rates of major bleeding in selected thrombocytopenic patients, were also conducted well above the values encountered here (7–9). Neither extrapolates directly to this scenario.

This report describes an unusual case in which urgent dual-lumen PICC placement was required before allogeneic HCT despite extreme thrombocytopenia and a poor sustained platelet increment. The last recorded count before the procedure was 2 × 10^9^/L; because a second transfusion was given immediately beforehand, the value at venipuncture was not remeasured, and this is stated wherever the case is described. The contribution is a transparent account of a nursing-led risk-control pathway, together with the measurements that would strengthen such reports and practical means of securing them elsewhere under comparable time pressure.

## Case description

A 56-year-old woman was admitted to a protected hematology transplantation unit for allogeneic HCT preparation. She had a diagnosis of SAA and concurrent fungal maxillary sinusitis. Her main bleeding manifestation was scattered ecchymosis for more than 3 months, graded retrospectively as World Health Organization grade 1 (10); she had no active epistaxis, gingival bleeding, gastrointestinal bleeding, hematuria, neurologic symptoms, or puncture-site bleeding on admission. She was afebrile, hemodynamically stable, pain-free, without palpable splenomegaly, and independent in daily activities. Mild situational anxiety was identified during nursing assessment.

Relevant history included gastric polyps without active treatment. She had received repeated red-cell and platelet transfusions during the preceding 3 months, so alloimmunization could not be excluded. No smoking or alcohol use, known allergy, or previous transfusion reaction was documented. Initial testing showed platelets 1 × 10^9^/L, hemoglobin 65 g/L, leukocytes 2.63 × 10^9^/L, absolute neutrophil count 0.59 × 10^9^/L, and absolute reticulocytes 8 × 10^9^/L. Prothrombin time, activated partial thromboplastin time, and fibrinogen were normal. Bone marrow examination showed markedly reduced hematopoiesis with nucleated cells below 5%; immunophenotyping, cytogenetics, flow cytometry for a paroxysmal nocturnal hemoglobinuria clone, and a gene panel showed no abnormality. The marrow findings, with platelets and reticulocytes both below 20 × 10^9^/L, met the Camitta criteria for severe rather than very severe disease (2, 11).

The planned conditioning regimen included busulfan, cyclophosphamide, and antithymocyte globulin. Antithymocyte globulin required a dedicated channel for prolonged infusion and busulfan had to run uninterrupted on a fixed pharmacokinetic schedule, so the concurrent transfusion, hydration, antifungal therapy, and parenteral support required could not have been delivered through one lumen without interrupting time-critical infusions or adding peripheral punctures. A dual-lumen device was therefore necessary; a 4-Fr single-lumen catheter was rejected for that reason, and no dual-lumen power-injectable device smaller than 5 Fr was held in the institutional inventory. Deferral was also rejected: donor collection and graft infusion dates were fixed, invasive fungal sinusitis required uninterrupted antifungal therapy, and the first transfusion gave no expectation that waiting would raise the count.

A short peripheral catheter was inadequate for sustained multi-channel treatment, and a midline catheter could not provide the central tip required for vesicant therapy. Subclavian access was judged least acceptable because that site is not compressible. Internal jugular access was appraised separately rather than grouped with it: although compressible and equally amenable to ultrasound guidance, its exit site lies close to an actively infected paranasal sinus and to an oral cavity expected to develop mucositis, neck dressings are hard to maintain, and a hematoma there can compromise the airway. Femoral access was unsuitable during a prolonged admission in a neutropenic, ambulant patient. Ultrasound-guided PICC placement through a compressible upper-arm vein was therefore selected (Table 1).

**Table 1:** Timeline of the episode of care.

Time/interval	Clinical event and intervention	Outcome signal
Hospital day 0	Admission to protected hematology unit; baseline nursing and laboratory assessment	Platelets 1 × 10^9^/L; no active bleeding; urgent HCT preparation initiated
Hospital day 0	Temporary peripheral access; first 200-mL ABO-identical, leukocyte-reduced, irradiated single-donor apheresis platelet unit administered	No transfusion reaction documented
Hospital day 1, morning	Repeat blood count approximately 14 h after the transfusion ended; multidisciplinary access review	Platelets 2 × 10^9^/L; poor sustained increment; corrected count increment not calculable; central access still indispensable
Hospital day 1, pre-insertion	Second 200-mL apheresis platelet transfusion completed 15 min before skin preparation; rescue and compression resources prepared	Insertion followed the transfusion without an interval; the last recorded count of 2 × 10^9^/L precedes the second unit
Hospital day 1	Ultrasound-guided right basilic-vein PICC insertion	First-puncture success; WHO grade 1 oozing controlled by 10 min of manual compression; tip confirmed in the lower third of the SVC
First 24 h	Protocol-defined surveillance every 15 min for 1 h, every 30 min for 2 h, hourly to 8 h, and 4-hourly to 24 h	No WHO grade ≥2 bleeding, hematoma, allergy, migration, leakage, or occlusion
Inpatient period	Aseptic maintenance, dedicated lumen use, infection and thrombosis surveillance	No documented CR-BSI, clinically evident thrombosis, or device failure
Post-transplant day +12	Continued HCT supportive care	Hematopoietic recovery documented
Hospital day 42	Discharge education and home PICC-care plan	Discharged after meeting clinical criteria, with the catheter in situ
Catheter day 63	Elective PICC removal in the outpatient vascular-access clinic after completion of intravenous therapy	Catheter intact on removal; no bleeding, hematoma, or venous obstruction
3-month follow-up	Telephone and outpatient review	No reported catheter complication; recovery and independent function sustained

### Diagnostic assessment

Acquired SAA had been diagnosed by the treating hematology team before transplantation, using the Camitta criteria set out above with exclusion of alternative marrow disorders. The immediate procedural assessment focused on whether central access was indispensable, whether a lower-risk alternative could provide equivalent therapy, and whether modifiable bleeding and infection risks could be controlled.

The platelet count of 1 × 10^9^/L was a critical value on admission. A temporary left-hand peripheral catheter was used to administer 200 mL of ABO-identical, leukocyte-reduced, irradiated single-donor apheresis platelets (approximately 2.5 × 10^11^ platelets, 2 days old). The next sample was drawn about 14 h after the transfusion ended and showed 2 × 10^9^/L. Because that interval falls outside both the 10–60-min and the 18–24-h windows used for standardized assessment, no corrected count increment could be calculated, and the findings therefore indicate a poor sustained increment rather than established platelet transfusion refractoriness. She was afebrile with no splenomegaly and no evidence of disseminated intravascular coagulation, though repeated transfusion exposure made alloimmunization plausible. HLA and HPA antibody screening, platelet crossmatching, and HLA-selected units were not available within the urgent procedural window, so the mechanism of the poor response is not classified (12).

**Figure 1:**
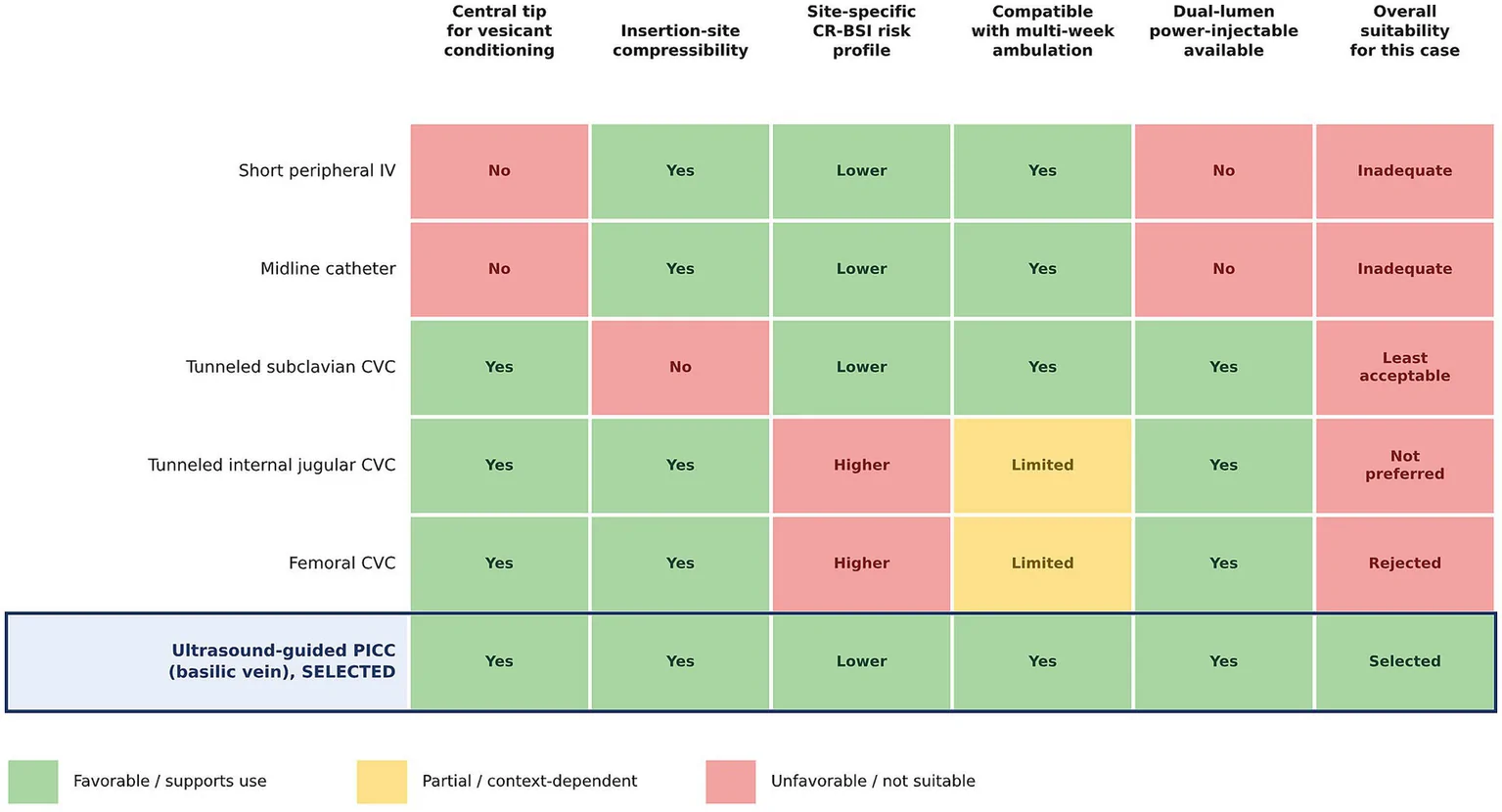
Comparative appropriateness of vascular-access routes considered for this case. The matrix summarizes the case-specific reasoning used by the multidisciplinary team. Internal jugular and subclavian routes are displayed separately because they differ in compressibility, in the feasibility of external hemostasis, and in site-care burden. Green indicates a favorable feature, amber a context-dependent feature, and red an unfavorable feature. It is not intended as a generalizable scoring tool. CR-BSI, catheter-related bloodstream infection; CVC, central venous catheter; IJ, internal jugular; SCV, subclavian; PICC, peripherally inserted central catheter.

The decision to proceed was conditional on hemodynamic stability, absence of active mucosal or neurologic bleeding, normal coagulation tests, an experienced operator, ultrasound-guided access at a compressible site, immediate compression and rescue resources, and capacity for high-frequency monitoring. A second platelet transfusion was ordered to precede the procedure, and insertion followed without an interval, so the last recorded count precedes the second unit and the value at venipuncture was not remeasured. This is stated wherever the case is described so that the observation can be weighed accordingly. Figure 1 summarizes the comparison of access alternatives.

### Therapeutic intervention

A second 200-mL unit of ABO-identical, leukocyte-reduced, irradiated single-donor apheresis platelets was administered on hospital day 1 over 40 min and completed 15 min before skin preparation. Physician-ordered premedication, controlled infusion, bedside observation, and immediate availability of rescue medication followed the institutional transfusion protocol then in force. Premedication was given to a patient without a prior transfusion reaction and is reported as institutional practice rather than as an evidence-based component of the pathway.

PICC insertion followed immediately, with the patient supine and the right arm abducted approximately 90 °. Ultrasound identified the right basilic vein as the preferred compressible vessel; measurement of the stored procedural images showed a transverse diameter of 5.2 mm, and with a 5-Fr outer diameter of 1.67 mm the catheter-to-vein ratio was approximately 32%, below the 45% ceiling recommended for peripherally inserted devices (13, 14). Maximal sterile barrier precautions and aseptic skin preparation were used. The catheter was inserted by the first author (YH), a PICC-credentialed hematology nurse who performs this procedure routinely in the unit, using ultrasound-guided modified Seldinger technique.

Venous access was obtained on the first puncture. Puncture-site oozing corresponding to World Health Organization grade 1 was controlled by 10 min of continuous manual compression, after which a pressure dressing was maintained for 24 h and bleeding did not recur. There was no hemodynamic instability, dyspnea, chest discomfort, arrhythmia, allergic reaction, expanding hematoma, or bleeding of grade 2 or higher. Postero-anterior chest radiography showed the tip 2.5 cm below the carina, within the lower third of the superior vena cava and approximately 1.5 cm above the cavoatrial junction; intracavitary electrocardiographic tip location was unavailable at our center. Conditioning therapy began after catheter function was confirmed.

One lumen was preferentially assigned to conditioning chemotherapy and the second to blood products, anti-infective therapy, and parenteral support. Maintenance included pulsatile flushing, positive-pressure locking, aseptic hub care, transparent-dressing surveillance, external-length checks, and prompt dressing replacement. The patient and family were taught to avoid traction, compression, and contamination, and to report bleeding, dressing saturation, leakage, resistance, fever, arm pain, or swelling.

The first 24 h were treated as the highest-risk period for bleeding and early device instability. Surveillance intervals were defined in advance: every 15 min for the first hour, every 30 min for the next 2 h, hourly until 8 h, every 4 h until 24 h, and once each nursing shift thereafter until removal. Each assessment covered oozing, dressing saturation, ecchymosis, hematoma, arm swelling, pain, upper-arm circumference, leakage, external catheter length, and blood return. Systemic monitoring covered vital signs, neurologic status, mucosal bleeding, urine color, and stool appearance. Tables 2, 3 summarize the procedure and the risk-control pathway with its basis and escalation thresholds.

**Table 2:** PICC procedure characteristics and immediate outcome.

Item	Documented detail	Case-specific interpretation
Platelet support before insertion	Two 200-mL apheresis units; the second completed 15 min before skin preparation	Last recorded count 2 × 10^9^/L, obtained before the second unit; insertion followed without an interval, so this value precedes the transfusion effect
Device	5-Fr dual-lumen power-injectable PICC (outer diameter 1.67 mm)	Dual channel required for uninterrupted conditioning with concurrent supportive therapy; no smaller dual-lumen power-injectable device available
Access site	Right basilic vein, transverse diameter 5.2 mm	Compressible upper-arm site; catheter-to-vein ratio approximately 32%, below the recommended 45% ceiling
Technique	Ultrasound-guided modified Seldinger technique performed by the first author (YH), a PICC-credentialed hematology nurse	Direct visualization and reduced microtrauma; the operator is a listed author, as disclosed in the limitations
Puncture attempts	One	Prespecified first-pass procedural goal achieved
Catheter measurements	Internal length 36 cm; external length 0 cm	Baseline for position and migration surveillance
Upper-arm circumference	25 cm at insertion	Baseline for swelling and thrombosis surveillance
Tip position	Lower third of the superior vena cava, 2.5 cm below the carina and approximately 1.5 cm above the cavoatrial junction on postero-anterior chest radiograph	Central use permitted after confirmation; intracavitary electrocardiographic tip location not available
Immediate outcome	WHO grade 1 oozing controlled by 10 min of manual compression and a 24-h pressure dressing; no grade ≥2 bleeding, expanding hematoma, allergy, or instability	Procedure completed without immediate serious complication

**Table 3:** Risk-control pathway, escalation triggers, and observed outcomes.

Risk domain	Controls, basis, and escalation thresholds	Observed outcome
Hemorrhage	Basis: guideline-supported (5, 13, 16). Platelet support before the procedure; avoidance of nonessential punctures; ultrasound-guided first-pass technique; 10 min of planned manual compression followed by a 24-h pressure dressing; scheduled site and systemic bleeding checks at the intervals defined in the text. Escalate for oozing persisting beyond 30 min of compression, saturation of more than half the dressing, ecchymosis extending more than 2 cm beyond its marked border, any neurologic change, hematuria, melena, or hemodynamic instability.	No WHO grade ≥2 insertion-site or spontaneous bleeding documented over 63 catheter-days
Transfusion reaction	Basis: institutional transfusion protocol; premedication is reported as local practice rather than as an evidence-based component. Controlled infusion over 40 min; bedside observation throughout and for 30 min afterward; rescue medication and equipment at the bedside. Escalate for rash, dyspnea, hypotension, a temperature rise of 1°C or more, chills, or acute deterioration.	No documented transfusion or periprocedural allergic reaction
Infection	Basis: guideline-supported (17, 18). Maximal sterile barrier precautions; hub disinfection for 15 s before every access; transparent dressing reviewed at every scheduled check and changed weekly or sooner if compromised; temperature recorded 4-hourly. Escalate for temperature of 38.0°C or higher, local inflammation, purulence, or unexplained deterioration.	Brief fever investigated; no local catheter signs or documented CR-BSI
Thrombosis	Basis: guideline-supported assessment (13, 14); surveillance was clinical rather than imaging-based. Catheter-to-vein ratio documented at insertion; baseline and daily upper-arm circumference measured 10 cm above the antecubital fossa; traction and compression avoided. Escalate for a circumference increase of 2 cm or more, arm pain, venous distension, or catheter dysfunction.	No clinically evident thrombosis over 63 catheter-days; surveillance was clinical, so asymptomatic thrombosis was not assessed
Mechanical failure	Basis: guideline-supported (13). Sutureless securement; external-length check at every scheduled assessment; pulsatile flushing and positive-pressure locking after each use; dressing integrity review. Escalate for any change in external length, leakage, resistance to flushing, absent blood return, kinking, or fracture.	No documented migration, leakage, occlusion, fracture, or infusion failure
Treatment continuity	Basis: guideline-supported (13, 16). Radiographic tip confirmation before first use; preferential lumen assignment; documented compatibility check before every co-infusion.	Conditioning began after confirmation; no access-related treatment delay

### Follow-up and outcomes

During the first 24 h the dressing remained stable, external catheter length remained 0 cm, and both lumens maintained blood return. After the initial grade 1 oozing was controlled, no further puncture-site bleeding, expanding hematoma, gross hematuria, melena, acute neurologic sign, fever, or allergic reaction was documented.

The PICC remained the primary central access route during conditioning, transplantation, and supportive therapy. Structured daily maintenance documentation for 27 consecutive inpatient days recorded an intact site, preserved catheter function, and no local inflammation, leakage, fracture, migration, or occlusion; the remaining days were covered by routine shift entries without recorded complication. A brief febrile episode occurred early after conditioning. The insertion site remained clinically normal, paired blood cultures were negative, and no catheter-related bloodstream infection was documented.

Arm circumference and symptoms were monitored for catheter-related thrombosis, and none was clinically evident. Surveillance was clinical rather than imaging-based, so asymptomatic thrombosis was not assessed, and absence of evidence should not be read as evidence of absence. Conditioning and stem-cell infusion proceeded without access-related delay. Hematopoietic recovery was documented on post-transplant day +12 (15). The patient was discharged after 42 days with the catheter *in situ*.

The catheter was retained for outpatient supportive therapy and electively removed in the vascular-access clinic 21 days after discharge, giving 63 catheter-days in total. It was intact on removal, with no bleeding, hematoma, or venous obstruction. At 3-month telephone and outpatient follow-up, no bleeding, fever, local infection symptom, or arm swelling was documented, and the records described sustained hematologic recovery, preserved organ function, donor chimerism consistent with engraftment, and independent daily functioning.

## Discussion

This case is unusual because indispensable central access was obtained immediately after platelet transfusion in a patient whose last recorded count was 2 × 10^9^/L, far below the range evaluated in randomized procedural evidence. Insertion followed the transfusion without an interval, so the value at venipuncture was not remeasured, and the observations are framed accordingly throughout. What the case offers is an explicit description of the reasoning, controls, procedural detail, and surveillance applied; a single favorable outcome cannot establish safety at this platelet level.

The randomized trial by van Baarle and colleagues evaluated prophylactic platelet transfusion before catheter placement at counts of 10–50 × 10^9^/L (6), and PICC-specific studies have extended experience below 20 × 10^9^/L and to an institutional threshold of 10 × 10^9^/L, but none addresses single-digit counts (7–9). This report does not propose a new device, technique, or transfusion strategy, and a single observation cannot establish safety. Its contribution is granular documentation at the extreme lower margin of a thin evidence base, in a form a prospective series could adopt directly.

The poor sustained increment influenced the decision pathway, since an apheresis unit would usually produce a substantial early rise in a responsive adult. Because the only available count was drawn approximately 14 h after the transfusion and no HLA or HPA testing was performed, the response is not assigned to a specific mechanism. These data indicate a poor sustained increment; they do not support a diagnosis of refractoriness.

The measurements that were unavailable in this case are, in most centers, obtainable without delaying an urgent procedure, and this is the case’s most transferable lesson. A corrected count increment requires only a count drawn 10–60 min after the transfusion ends, the platelet dose on the component label, and the patient’s body surface area; the sample can be drawn from the existing peripheral line while the sterile field is prepared, and an automated result is usually available within 20 min. Antibody screening is harder to arrange at short notice, but transplant candidates have already been HLA typed for donor matching, so the tissue-typing laboratory generally holds a stored sample and an add-on class I screen can be requested during the work-up rather than at the moment of crisis, with a registry request for HLA-selected units running in parallel (12).

A PICC offered direct ultrasound visualization, a compressible insertion site, and a central tip while avoiding a neck, chest, or groin puncture. Internal jugular and subclavian access differ materially and were therefore appraised separately: the subclavian site is not compressible whereas the internal jugular site is, and the two differ further in site-care burden and in the consequences of a local hematoma. These advantages do not eliminate risk, since PICCs can cause thrombosis, occlusion, infection, and migration, particularly with larger or multi-lumen catheters in hematology populations (13, 14, 16–19). The catheter-to-vein ratio of approximately 32% was derived from the stored procedural images, measured in a single transverse plane without standardized tourniquet conditions.

Several controls converged on the insertion event, and which was decisive cannot be identified; the pathway should be read as a bundle rather than as evidence for any single maneuver.

Infection prevention was a parallel priority in a patient with neutropenia, active fungal sinusitis, conditioning-related mucosal injury, and a central catheter. Maximal sterile barrier precautions, aseptic access, dressing integrity, hub disinfection, and daily assessment of infection signs were maintained; the transient fever was investigated without local catheter findings.

Several limitations should be borne in mind. Insertion followed the second transfusion without an interval, so the value at venipuncture was not remeasured and the report describes a decision pathway rather than a platelet threshold. The sampling interval also precluded a corrected count increment, so the poor increment is not assigned a mechanism and refractoriness is neither confirmed nor excluded. Thrombosis surveillance was clinical rather than imaging-based, so asymptomatic thrombosis was not assessed, and absence of evidence is not evidence of absence. The catheter-to-vein ratio was derived from the stored images rather than recorded prospectively. The report describes a single patient, the data derive from routine clinical documentation rather than a prespecified protocol, bleeding was graded retrospectively against the World Health Organization scale, and the operator is one of the authors, which is disclosed for transparency. Finally, no comparison with an alternative catheter, delayed insertion, or a different platelet product is possible.

The take-away lesson is practical. When central access is indispensable and delay carries its own substantial harm, the decision can be made explicit rather than implicit: necessity and alternatives appraised by a multidisciplinary team, a compressible ultrasound-accessible vessel chosen with a documented catheter-to-vein ratio, an experienced operator committed to a first-pass strategy, and monitoring intervals and escalation thresholds defined before the needle is introduced. This report documents that pathway in full and specifies the variables a prospective multicenter series would need to record. A single case cannot establish the safety of PICC placement at extremely low platelet counts or define a platelet threshold.

## Patient perspective

The patient reported that admission to a protected environment and the seriousness of the diagnosis initially caused anxiety. She understood that the catheter was needed for conditioning, transfusions, and supportive care, and that her bleeding risk was unusually high. Structured explanation of the procedure, family involvement when permitted, and relaxation techniques reduced her anxiety. After discharge she reported confidence in protecting the catheter at home and a gradual return to independent daily activities.

## CARE reporting

This case report was prepared in accordance with the CARE guidelines (20, 21). A completed CARE checklist is provided as Supplementary material.

## Data Availability

The raw data supporting the conclusions of this article will be made available by the authors, without undue reservation.
